# An Extremely Premature Neonate with Severe Anemia

**DOI:** 10.21699/jns.v6i2.547

**Published:** 2017-04-15

**Authors:** Swosti Joshi, Ivy Mulinge, Medha Kamat

**Affiliations:** John H Stroger Jr. Hospital of Cook County Chicago, Illinois

**Keywords:** Neonate, Premature, Anemia, Splenic rupture

## Abstract

Spleen rupture in an extremely premature newborn is very rare event. High index of suspicion is required to make timely diagnosis and thereafter appropriate management. We present a rare case of an extremely premature, extremely low birthweight newborn who presented with severe anemia secondary to splenic rupture. It was managed conservatively without splenectomy resulting in complete resolution of symptoms. Although non-operative management of pediatric splenic injuries is now recognized as the treatment of choice, there is very little experience in premature newborns.

## Case Report

A female neonate with birth weight 710 grams was born to a 20 years old Gravida 2 Para 0010 mother at 24 weeks of gestation by caesarean section done for preterm labor with breech presentation. The mother had unremarkable prenatal course. Only one dose of betamethasone was given about two hours prior to delivery. APGAR scores were 6 and 8 in 1 and 5 minutes of life respectively. After initial resuscitation, the neonate was placed in mechanical ventilator and was brought to neonatal intensive care unit. 

Physical examination was unremarkable. Anterior fontanel was at level. Normal heart sounds were normal with no murmur. The patient was intubated and had equal breath sounds bilaterally. Abdomen was soft and liver and spleen could not be palpated. Both the lower legs had some bruising however no other deformities were noted. Laboratory evaluation revealed hemoglobin level of 13-g/dL and total white blood cell count of 8.7 K/UL and Platelets170 k/UL. The neonate remained stable with usual neonatal course. At about 16 hours of life, hemoglobin level dropped to 8.8 g/dl. Despite multiple blood transfusions, hemoglobin level of the patient continued trending downwards to 7.8 g/dl and further to 5.8 g/dl. She required 110ml/kg of packed red cell transfusion in first 24 hours of life. She then required about 70ml/kg of packed red blood cells transfusion thereafter for next 24 hours. Meanwhile the neonate remained hemodynamically stable and did not require any vasopressor support. On second day of life, the platelet count decreased to 63k/ul and platelet transfusion was given. Platelet count thereafter remained stable. Anterior fontanel was full however not bulging. On auscultation, the patient was noted to have grade 2 soft systolic murmur. Abdomen was soft and mildly distended with bluish discoloration. After receiving another 40 ml/kg of packed red blood cell transfusion, the hemoglobin of the patient stabilized after 72 hours of life.

**Figure 1:**
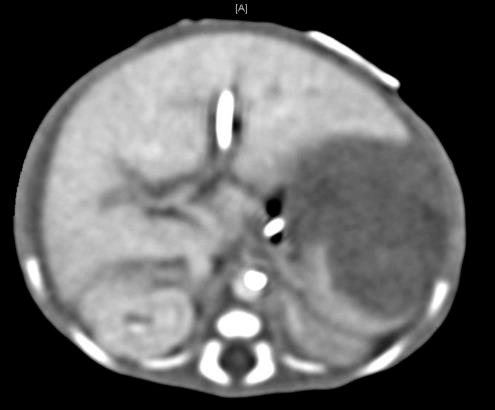
Computerized tomographic scan with IV con-trast of abdomen.

To further explore the cause of massive blood transfusion requirement in this extremely premature neonate, ultrasonography of head was done which showed bilateral germinal matrix bleed. Abdominal ultrasonography showed peritoneal fluid collection with normal hepatic texture however with irregular spleen. Computerized tomography of abdomen showed large sub capsular splenic hematoma with compressive effect on spleen (Fig.1). Peritoneal fluid collection was also seen which could be due to leaking from sub capsular splenic hematoma. As the patient was hemodynamically stable, surgical exploration was not done. The patient was given multiple transfusions of fresh frozen plasma. Coagulation profile, infection workup and skeletal survey were unremarkable. Subsequent ultrasonography showed the gradual resolution of splenic hematoma. The patient thereafter had uneventful recovery.

## Discussion:

Splenic injury in an extremely premature newborn is very rare event. Less than 50 cases of splenic rupture in newborn have been reported worldwide.[1] Various etiologies have been reported as causes of splenic bleed. The most common cause of bleeding is trauma, which can be due to precipitous delivery or difficult delivery.[2-4] The possibility of breech extraction could be the cause in this case. Besides trauma, underlying coagulation disorder could also lead to splenic bleed. There have been a number of case reports of idiopathic splenic bleeding as well.[5,6] A classic presentation of splenic rupture is a triad of bleeding, abdominal distension and hemoperitoneum. Due to high incidence of post-splenectomy sepsis, splenectomy is no longer considered the standard of treatment. [1] Achieving hemostasis with preservation of spleen is the mainstay of treatment. 

This is probably the first reported case of an extremely premature newborn diagnosed with splenic capsular hematoma treated without splenectomy. The patient did not present with classic triad. The neonate was delivered by caesarean section done for breech presentation. The first symptom began at 16 hours of life when patient was noted be anemic requiring red blood cell transfusion. Within next 24 hours the patient required about 110ml/kg of red blood transfusion. The neonate had mild abdominal distension but no other overt signs of intra-abdominal bleeding. Abdominal ultrasonography and CT scan of abdomen revealed capsular bleeding with intra-peritoneal leakage of blood. The baby was managed conservatively and saved without splenectomy. The recovery was uneventful. 

A patient may not present with classic presentation. But with high index of suspicion, the case can be diagnosed early and can be treated conservatively even in an extremely premature neonate.

## Footnotes

**Source of Support:** None

**Conflict of Interest:** None
